# Supplementary figures and images for: Cross-platform normalization of microarray and RNA-seq data for machine learning applications
Source: PeerJ. 2016 Jan 21;4:e1621. doi: 10.7717/peerj.1621 (PMC4736986; doi:10.7717/peerj.1621)

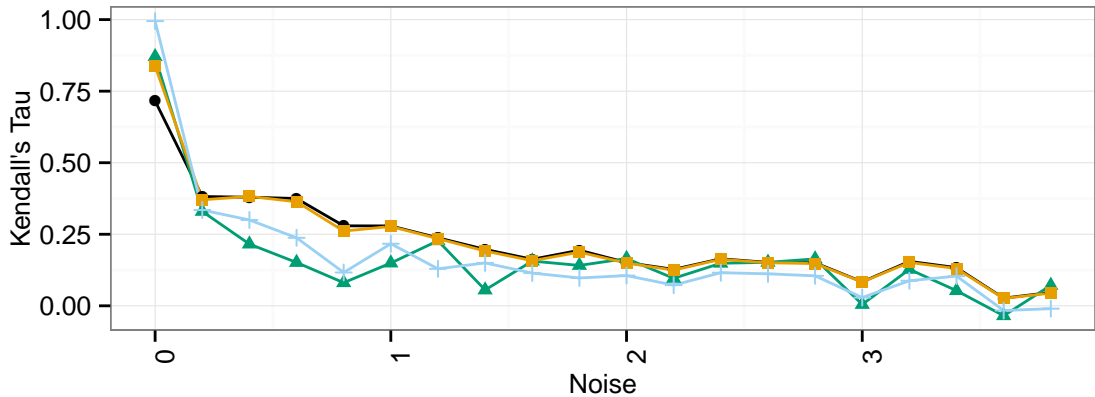

Dataset

- TDM
- LOG
- QN
- NPN

Supplement: Supplemental Information 1 — Average Kendall’s tau across 20 levels of noise in the simulated data. [file peerj-04-1621-s001.pdf]

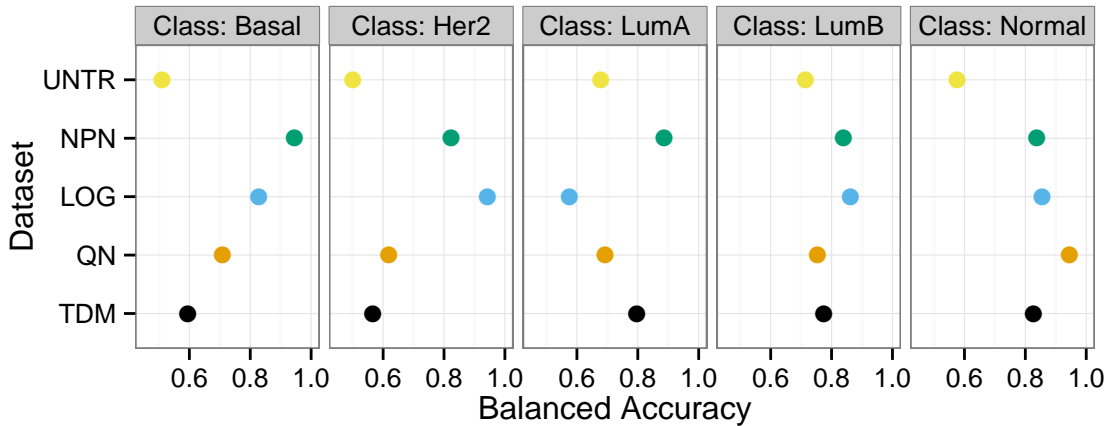

Supplement: Supplemental Information 2 — Average balanced accuracy for BRCA subtype classification by subtype using TCGA microarray training data and TCGA RNA-seq test data. [file peerj-04-1621-s002.pdf]

**A**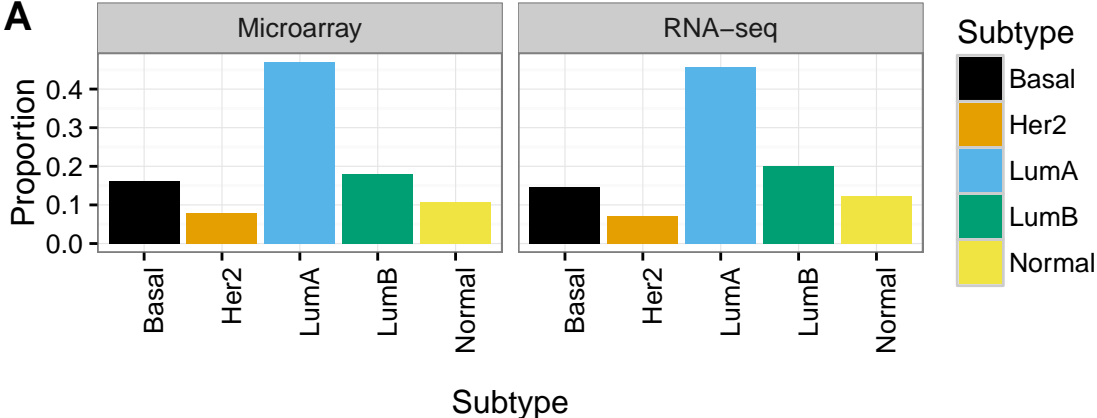**B**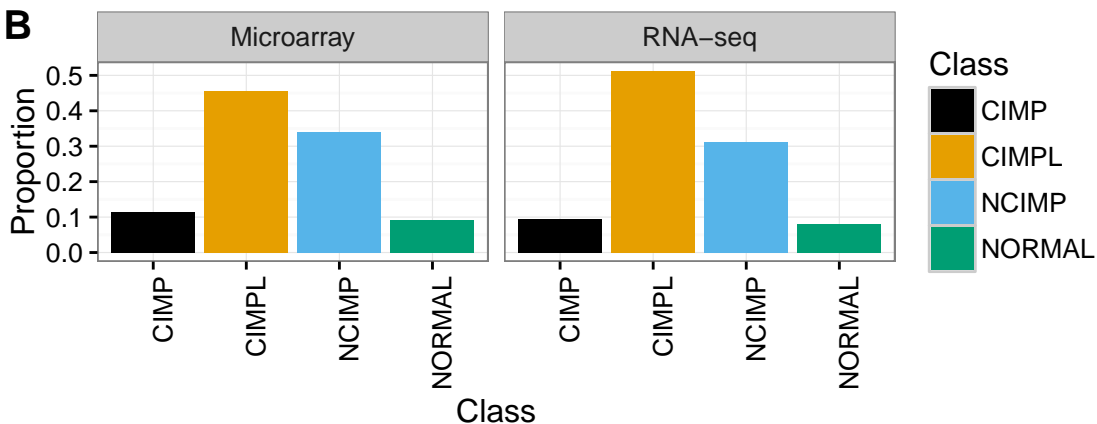**C**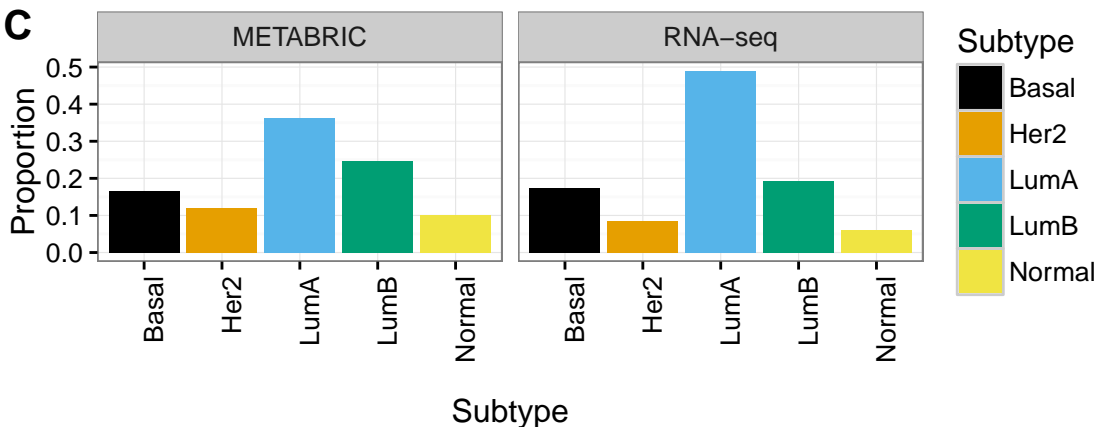

Supplement: Supplemental Information 3 — The distribution of classes in the data is roughly the same between each training and testing set. (A) Distribution of classes for the first biological dataset, using TCGA microarray of breast cancer biopsies for training and TCGA RNA-seq for testing. (B) Distribution of classes for the second biological dataset, using TCGA microarray of colon/rectal cancer biopsies for training and TCGA RNA-seq for testing. (C) Distribution of classes for the third biological dataset, using METABRIC microarray of breast cancer biopsies for training and TCGA RNA-seq for testing. [file peerj-04-1621-s003.pdf]

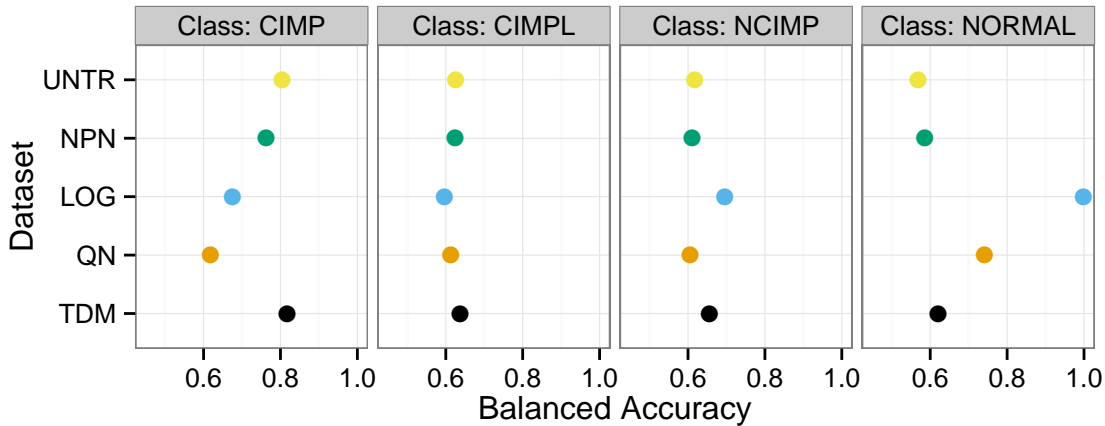

Supplement: Supplemental Information 4 — Average balanced accuracy for colon/rectal cancer CIMP classification using TCGA microarray for training and TCGA RNA-seq for test data. [file peerj-04-1621-s004.pdf]

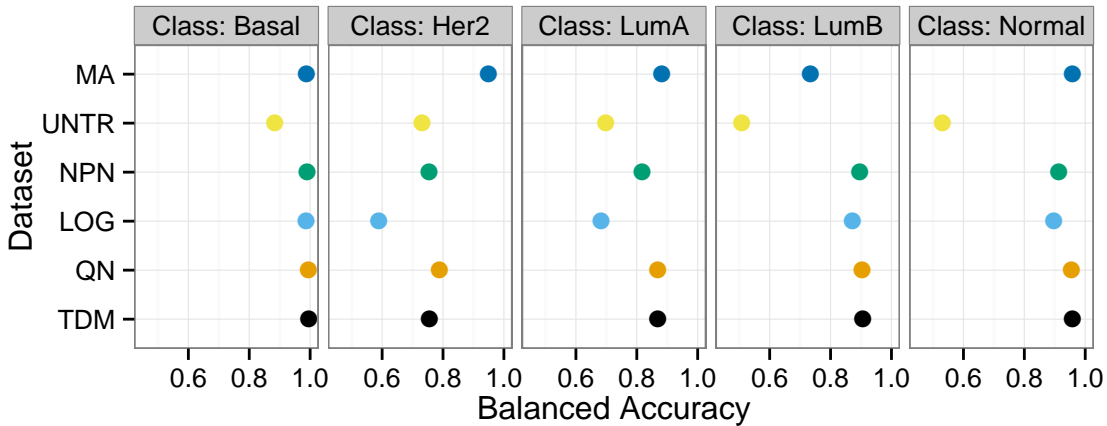

Supplement: Supplemental Information 5 — Average balanced accuracy for BRCA subtype classification by subtype using METABRIC microarray training data and TCGA RNA-seq test data as well as TCGA microarray test data for comparison. [file peerj-04-1621-s005.pdf]
